# Artificial Intelligence-Enhanced Echocardiography for Systolic Function Assessment

**DOI:** 10.3390/jcm11102893

**Published:** 2022-05-20

**Authors:** Zisang Zhang, Ye Zhu, Manwei Liu, Ziming Zhang, Yang Zhao, Xin Yang, Mingxing Xie, Li Zhang

**Affiliations:** 1 Department of Ultrasound Medicine, Union Hospital, Tongji Medical College, Huazhong University of Science and Technology, Wuhan 430022, China; m202176005@hust.edu.cn (Z.Z.); zhuye_@hust.edu.cn (Y.Z.); liumw411@hust.edu.cn (M.L.); zzm9936@hust.edu.cn (Z.Z.); m202176093@hust.edu.cn (Y.Z.); 2 Clinical Research Center for Medical Imaging in Hubei Province, Wuhan 430022, China; 3 Hubei Province Key Laboratory of Molecular Imaging, Wuhan 430022, China; 4 Media and Communication Lab (MC Lab), Electronics and Information Engineering Department, Huazhong University of Science and Technology, Wuhan 430022, China; xinyang2014@hust.edu.cn

**Keywords:** echocardiography, artificial intelligence, left ventricular systolic function, machine learning, deep learning

## Abstract

The accurate assessment of left ventricular systolic function is crucial in the diagnosis and treatment of cardiovascular diseases. Left ventricular ejection fraction (LVEF) and global longitudinal strain (GLS) are the most critical indexes of cardiac systolic function. Echocardiography has become the mainstay of cardiac imaging for measuring LVEF and GLS because it is non-invasive, radiation-free, and allows for bedside operation and real-time processing. However, the human assessment of cardiac function depends on the sonographer’s experience, and despite their years of training, inter-observer variability exists. In addition, GLS requires post-processing, which is time consuming and shows variability across different devices. Researchers have turned to artificial intelligence (AI) to address these challenges. The powerful learning capabilities of AI enable feature extraction, which helps to achieve accurate identification of cardiac structures and reliable estimation of the ventricular volume and myocardial motion. Hence, the automatic output of systolic function indexes can be achieved based on echocardiographic images. This review attempts to thoroughly explain the latest progress of AI in assessing left ventricular systolic function and differential diagnosis of heart diseases by echocardiography and discusses the challenges and promises of this new field.

## 1. Left Ventricular Systolic Function Assessment in Clinical Practice

Left ventricular systolic dysfunction is a common disorder mainly caused by myocarditis, cardiomyopathy, and ischemic heart diseases [1,2]. It gives rise to fatigue, dyspnea, or even death from heart failure [1,2]. Accurate assessment of left ventricular systolic function is crucial for the diagnosis, treatment, and prognosis of cardiovascular diseases.

Currently, parameters such as LVEF, GLS, and peak systolic velocity of the mitral annulus (S’) are used to evaluate left ventricular systolic function in clinical practice [1,2,3,4,5]. Echocardiography has become the mainstay of cardiac imaging in measuring LVEF and GLS as it is non-invasive, radiation-free, and easy to obtain. Although echocardiography plays a vital role in the dynamic treatment and follow-up of cardiovascular diseases, there remain some limitations such as dependence on experience, intra-observer variability, and inter-observer variability. Recently, AI has shown increasing promise in medicine to overcome these challenges.

## 2. AI’s Application in Left Ventricular Systolic Function Assessment

### 2.1. Key Concepts in AI

Medical imaging has great significance in the diagnosis, treatment, and prognosis of diseases with the characteristics of a large amount of data and rich information of lesions. However, humans have a limited ability to interpret high-dimensional image features. As an effective tool, AI assists experts in analyzing medical imaging data, which can improve the accuracy and repeatability of complex and multi-dimensional data analysis [6,7].

**Figure 1:**
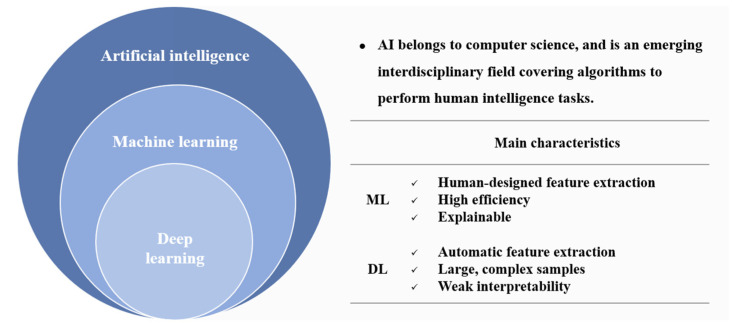
Logical diagram of AI, ML, and DL and the main characteristics of ML and DL. (AI: artificial intelligence; ML: machine learning; DL: deep learning).

AI is a field of computer science in which algorithms are used to perform human-intelligence tasks, and it is an emerging interdisciplinary field covering computers, psychology, and philosophy [8]. Machine learning (ML) is a subset of AI, and deep learning (DL) is an essential branch of ML [8] (Figure 1). ML uses statistical algorithms such as the random forest (RF) and support vector machine (SVM) algorithms to quickly, accurately, and efficiently analyze complex clinical data based on feature engineering. They establish decision-aided models for cardiovascular diseases with high efficiency and good model interpretability. However, machine learning requires human-designed feature extraction, which is time consuming. DL realizes automatic feature extraction and plays a vital role in analyzing large and complex samples, but its interpretability still needs improvement [8,9].

### 2.2. AI in Echocardiography

**Figure 2:**
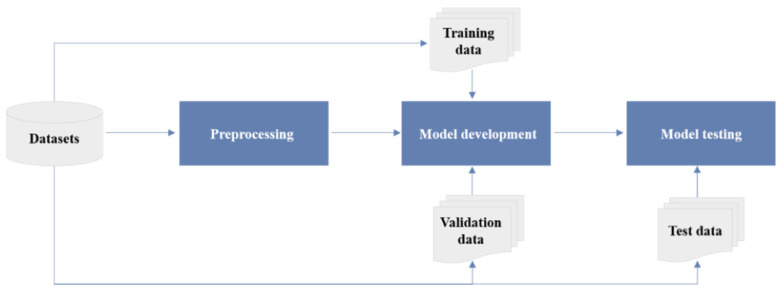
The workflow of studies on AI in echocardiography, including four main steps: (1) clinical problem-oriented data collection; (2) data preprocessing operations based on task characteristics (classification tasks require explicit sample labels; segmentation tasks require the marking of regions of interest) and data splitting (training, validation, and testing datasets are mutually independent); (3) based on the type of tasks (regression, classification, or clustering), appropriate AI algorithms are selected for model development on the training datasets, and the performance of the model is validated on the validation datasets; (4) the reliability and generalization of the model are tested on the internal and external independent testing datasets.

Currently, the research on AI’s application in echocardiography is proliferating. The series of studies follow a standard paradigm divided into four stages: data collection, preprocessing, model development, and model testing [10] (Figure 2).

**Figure 3:**
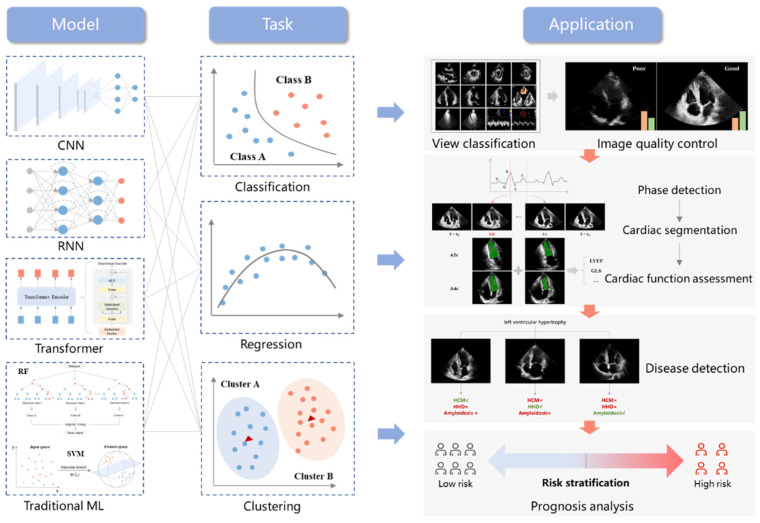
Overview diagram of AI’s application in echocardiography. Current applications focus on view classification and image quality control (classification), cardiac phase detection and cardiac function assessment (regression), and disease diagnosis and prognosis analysis (clustering). Mainstream AI algorithms consist of the convolutional neural network (CNN), recurrent neural network (RNN), transformer, and traditional machine learning algorithms (RF and SVM).

AI extracts features of ultrasound images to recognize standard views, segment cardiac structures, assess cardiac function, identify disease phenotypes, and analyze the prognosis combined with multi-dimensional parameters [7,11] (Figure 3).

View classification is the first step in real-time quantitative and post-processing cardiac structures and function analysis. Echocardiography consists of multimodal images, such as M-mode still images, two-dimensional gray-scale videos, and Doppler recordings [3]. Different modes contain multiple standard views [3]. Due to the between-subject variability and different imaging parameters, there are differences in the same standard view [3]. In addition, multiple standard views contain similar cardiac motion information (such as valvular motion and ventricular wall motion), increasing the challenge of view classification [3]. Several studies have confirmed the feasibility and accuracy of AI in analyzing echocardiography and classifying standard views [12,13,14,15]. AI algorithms represented by a convolutional neural network (CNN) can extract multi-scale features of ultrasonic images, improve the accuracy of view classification, and simplify the process of image processing. However, how to efficiently classify multimodal ultrasound images requires further studied for the high frame rate of echocardiography, small pixel changes between images, and information redundancy.

In terms of medical image processing, cardiac segmentation is the key to accurately assessing cardiac structures and function. Due to the complexity of heart movement and anatomic structures, edge blur, low signal-to-noise ratios, and small cardiac target regions, accurate heart segmentation based on echocardiography has always been an extremely challenging task [16]. AI algorithms are valuable for mining high-dimensional information that is not perceivable by the naked eye and for maximizing the extraction of image features. By integrating the spatial and temporal information, AI can identify critical cardiac anatomical structures, improve the accuracy of cardiac segmentation, and lay a solid foundation for the assessment of cardiac function [16,17,18].

Human assessment of cardiac function depends on the sonographer’s experience, and inter-observer and intra-observer variability exist [19]. Based on AI algorithms, fully automatic, comprehensive analyses of cardiac function can be achieved. Additionally, they also improve the accuracy and repeatability of image interpretation, simplify the diagnosis and treatment process, and reduce the time and labor costs, which have significant clinical value [19,20,21,22].

Disease diagnosis, differential diagnosis, and prognosis analysis usually require the integration of multi-dimensional imaging parameters and clinical information to build disease decision models. The traditional statistic methods require a priori knowledge by clinicians, and the prediction effect is poor when dealing with extensive sample data [23]. AI performs well in analyzing high-dimensional and complex data with strong feature extraction ability. It has significant advantages over clinicians in analyzing complex clinical information and realizing personalized risk stratification [23,24,25,26,27].

With the continuous development of ultrasound imaging and computer technology, the application of AI in the quantitative assessment of heart structures and function is shifting from single-frame images and two-dimensional video to three-dimensional images, from ML to DL, and from single-tasking to multitasking. The emergence of the latest AI algorithms has injected new vitality into quantifying cardiac function in echocardiography. These algorithms are expected to help achieve automatic full-stack analysis in echocardiography and improve the accuracy and reproducibility of cardiac function assessments. However, AI’s application in echocardiography is in its initial stage, and the current hot spot still focuses on automatic left ventricular systolic function assessment. This paper reviews the current studies on AI-enhanced echocardiography in evaluating left ventricular systolic function.

## 3. AI’s Application in Left Ventricular Systolic Function—LVEF

LVEF is one of the most critical indexes of cardiac systolic function. However, human assessment of cardiac function has inter-observer variability despite the sonographer’s years of training. Therefore, researchers have been working on a fully automated method of assessing LVEF (Table 1).

### 3.1. Cardiac Segmentation

Accurate segmentation of the left ventricle is the basis for estimating LVEF. In 2019, Leclerc S. et al. [16] made a large publicly available dataset called Cardiac Acquisitions for Multi-Structure Ultrasound Segmentation (CAMUS). It contained two- and four-chamber views of 500 patients with expert annotations. Multiple algorithms were adopted to segment structures such as the left ventricular endocardium, myocardium, and left atrium based on the dataset. Then they measured end-diastolic and end-systolic volumes and LVEF using Simpson’s biplane method. The results showed that the optimized AI model performed better than the other algorithms in segmenting the left ventricle. The automatic measurements of the left ventricular volumes and LVEF were consistent with expert assessments. In the same year, Smistad E. et al. [17] used the CAMUS dataset and an additional dataset of 106 patients with apical long-axis views to construct a multi-view segmentation network by transfer learning. It turned out that the network successfully segmented the left ventricle and left atrium in apical long-axis views. Later, Leclerc S. et al. [18] proposed a multistage attention network. Compared with the AI model mentioned above, this network was optimized by adding a region proposal network to locate the left ventricle before segmentation. The results showed that the network improved the accuracy and robustness of left ventricle segmentation.

Previous studies have focused mainly on the segmentation of the cardiac cavity in a single video frame. However, echocardiography provides rich information in the temporal domain. Therefore, Wei H. et al. [28] designed a network called Co-Learning of Segmentation and Tracking on Appearance and Shape Level (CLAS). It provides segmentation of the whole sequences with high temporal consistency and an accurate assessment of LVEF.

### 3.2. Automatic Assessment of LVEF

Based on the image segmentation, researchers have devoted themselves to developing fully automatic models to assess LVEF.

Ouyang et al. [19] presented a video-based AI model called EchoNet-Dynamic to evaluate cardiac function. The study included apical four-chamber views of 10,030 patients with annotations of only end-systole and end-diastole frames. They generalized these labels with a weak supervision approach and developed an atrous convolution model to generate frame-level semantic segmentation of the whole cardiac cycle. The weak supervision approach could cut the cost of labeling considerably. The atrous convolution model is able to integrate sufficient information into the temporal domain, provide frame-level segmentation of the left ventricle, and draw a left ventricular volume curve with multiple cardiac cycles to evaluate LVEF. The results showed that the EchoNet-Dynamic segmented the left ventricle accurately with a Dice similarity coefficient greater than 0.9, both at the end-systole and end-diastole levels, as well as across the cardiac cycle. The researchers further analyzed the accuracy of LVEF assessment by external validation of 2895 patients. It was revealed that the automatic measurements were consistent with the expert assessments and had good repeatability. In addition, the study discussed a series of problems that clinicians might experience in clinical practice, including the accuracy of model measurements of cardiac function in patients with arrhythmias and the measurements of different video qualities, using different instruments, and under different imaging conditions. The results showed that EchoNet-Dynamic was robust to variation in heart rhythm and video acquisition conditions.

Asch F.M. et al. [20] developed a computer vision model that estimated LVEF by simulating experts on a dataset of 50,000 studies. The results showed good correlation and consistency between automatic measurements and human assessments. In addition, Reynaud H. et al. [29] proposed a transformer model based on the self-attention mechanism, which can analyze echocardiographic videos of any length, locate ED and ES precisely, and assess LVEF accurately.

Zhang J. et al. [21] presented the first fully automated multitasking echocardiogram interpretation system to simplify the clinical diagnosis and treatment. The model successfully classified 23 views, segmented cardiac structures, assessed LVEF, and diagnosed three diseases (hypertrophic cardiomyopathy, cardiac amyloid, and pulmonary arterial hypertension). In 2022, Tromp J. et al. [30] developed a fully automated AI workflow to classify, segment, and interpret two-dimensional and Doppler modalities based on international and interracial datasets. The results showed that the algorithms successfully assessed LVEF with the area under the receiver operating characteristic curve (AUC) of 0.90–0.92.

The above studies discussed the quantification of LVEF in regular medical imaging practice. Nevertheless, Point-of-Care Ultrasonography (POCUS) is widely used in emergency and severe cases. POCUS is the acquisition, interpretation, and immediate clinical integration of ultrasound imaging performed by a clinician at the patient’s bedside rather than by a radiologist or cardiologist [31]. POCUS enables direct interaction between patients and clinicians, contributing to accurate diagnoses and treatments. Moreover, POCUS can provide timely diagnoses for emergency and critically ill patients as the ultrasonic device is easy to carry and handle. However, one of the prerequisites for using POCUS is that the clinician is competent in operating the device and interpreting the data obtained, which requires standardized training. To overcome this challenge, researchers have turned their attention to AI. In 2020, the FDA approved two products of Caption Health: Caption Guidance and Caption Interpretation. Caption guidance could successfully guide novices without ultrasonographic experience to obtain diagnostic views after short-term training [15]. Caption Interpretation was able to assist doctors in measuring LVEF automatically [20]. Then, they combined Caption Guidance and Caption Interpretation to develop a new AI algorithm setting in POCUS [32]. The algorithm allowed for image acquisition, quality assessments, and fully automatic measurements of LVEF, which could assist doctors in collecting and analyzing images accurately and quickly.

The above studies indicate that AI algorithms have essential clinical values: they can improve the accuracy of LVEF assessment, simplify the workflow of diagnosis and treatment, and reduce time and labor costs.

### 3.3. Disease Diagnosis

According to the 2021 ESC guidelines [1], heart failure is divided into three categories: heart failure with reduced ejection fraction (LVEF ≤ 40%), heart failure with mildly reduced ejection fraction (LVEF 41~49%), and heart failure with preserved ejection fraction (LVEF ≥ 50%). The three types of heart failure have different degrees of left ventricular systolic and diastolic dysfunction. However, there is no sensitive prediction model based on echocardiographic indicators to stage heart failure for patients with similar features or to predict major adverse cardiac events. For this, Tokodi M. et al. [24] applied topological data analysis (TDA) to integrate echocardiographic parameters of left ventricular structures and function into a patient similarity network. The TDA network can represent geometric data structures according to the similarity between multiple echocardiographic parameters, retain critical data features, and effectively capture topological information of high-dimensional data. The results revealed that the TDA network successfully divided patients into four regions based on nine echocardiographic parameters and performed well in the dynamic evaluation of the disease course and the prediction of major adverse cardiac events. Cardiac function gradually deteriorated from region one to region four.

It can be seen that AI algorithms perform well in mining the potential features of echocardiographic data, and hence facilitate early and accurate diagnosis.

**Table 1:** Studies of AI’s Application in Left Ventricular Systolic Function—LVEF.

Authors	Year	Task	Model	Dataset	Results
Leclerc S. et al. [16]	2019	LV segmentation	U-Net	500 subjects	Accuracy in LV volumes (MAE = 9.5 mL, r = 0.95).
Smistad E. et al. [17]	2019	LV segmentation	U-Net	606 subjects	Accuracy for LV segmentation (DSC 0.776–0.786).
Leclerc S. et al. [18]	2020	LV segmentation	LU-Net	500 subjects	Accuracy in LV volumes (MAE = 7.6 mL, r = 0.96).
Wei H. et al. [28]	2020	LV segmentation	CLAS	500 subjects	Accuracy for LVEF assessment (r = 0.926, bias = 0.1%).
Reynaud H. et al. [29]	2021	LVEF assessment	Transformer	10,030 subjects	Accuracy for LVEF assessment (MAE = 5.95%, R^2^ = 0.52).
Ouyang et al. [19]	2020	LVEF assessment	EchoNet-Dynamic	10,030 subjects	Accuracy for LV segmentation (DSC = 0.92), LVEF assessment (MAE = 4.1%), and HFpEF classification (AUC 0.97).
Asch F.M. et al. [20]	2019	LVEF assessment	CNN	>50,000 studies	AutoEF values show agreement with GT: r = 0.95, bias = 1.0%, with sensitivity 0.90 and specificity 0.92 for detection of EF less than 35%.
Zhang J. et al. [21]	2018	LVEF assessment GLS assessment Disease detection	CNN	14,035 studies	Agreement with GT: for LVEF, MAE = 9.7%; for GLS, and MAE = 7.5% and 9.0% (within 2 cohorts). Disease detection: HCM, Amyloid, and PAH (AUC 0.93, 0.87, and 0.85).
Tromp J. et al. [30]	2022	LVEF assessment	CNN	43,587 studies	Accuracy for LVEF assessment (MAE 6–10%).
Narang A. et al. [15]	2021	LVEF assessment	Caption Guidance	240 subjects	LV size, function, and pericardial effusion in 237 cases (98.8%) and RV size in 222 cases (92.5%) are of diagnostic quality.
Asch F.M. et al. [32]	2021	LVEF assessment	Caption Health	166 subjects (Protocol 1) 67 subjects (Protocol 2)	Protocol 1: agreement with GT: ICC 0.86–0.95, bias < 2%. Protocol 2: agreement with GT: ICC = 0.84, bias 2.5 ± 6.4%.
Tokodi M. et al. [24]	2020	Disease detection (HFpEF)	TDA	1334 subjects	Region 4 relative to 1: HR = 2.75, 95%CI 1.27–45.95, *p* = 0.01. Correlation of NYHA and ACC/AHA stages with regions: r = 0.56 and 0.67.

LVEF, left ventricular ejection fraction; LV, left ventricle; RV, right ventricle; MAE, mean absolute error; DSC, dice similarity coefficient; CLAS, Co-Learning of Segmentation and Tracking on Appearance and Shape Level; AUC, area under the receiver operating characteristic curve; CNN, convolutional neural network; GT, ground truth; GLS, global longitudinal strain; HCM, hypertrophic cardiomyopathy; PAH, pulmonary arterial hypertension; HFpEF, heart failure with preserved ejection fraction; HF, heart failure; ICC, intraclass correlation coefficient; TDA, topological data analysis; HR, hazard ratio; CI, confidence interval; NYHA, New York Heart Association; ACC, American College of Cardiology; AHA, American Heart Association.

## 4. AI’s Application in Left Ventricular Systolic Function—GLS

LVEF is one of the main indexes of cardiac systolic function. However, it is not sensitive for the identification of early ventricular systolic dysfunction. The guidelines published by the Heart Failure Association of the European Society of Cardiology (ESC) [33] pointed out that GLS was superior to LVEF in the evaluation of subclinical ventricular systolic dysfunction due to it being stable and repeatable. Therefore, the guidelines recommended using GLS to detect subclinical ventricular systolic dysfunction. The current challenge is that GLS post-processing is time-consuming. Hence, researchers have turned to AI to meet these challenges (Table 2).

### 4.1. Automatic Assessment of GLS

AutoStrain is an application integrated into the EPIQ CVx system that can measure GLS automatically. A study [34] showed that AutoStrain was feasible in 99.5% of patients. However, there was discordance between automated, semi-automated, and manual measurements (automated vs. manual GLS: r = 0.685, bias = 0.99%; semi-automated vs. manual GLS: r = 0.848, bias = −0.90%; automated vs. semi-automated GLS: r = 0.775, bias = 1.89%). Approximately 40% of patients needed manual correction after automated assessments. Therefore, it is necessary to strike a balance between the post-processing speed and accuracy of assessment so that automatic software can be widely used in clinical practice and assist doctors in evaluating patients’ myocardial motion quickly and accurately.

At present, automatic assessment is still based on speckle tracking echocardiography. It tracks the deformation of local speckles between two consecutive frames, which is easily affected by the signal-to-noise ratio of the images. Furthermore, post-processing is time consuming. Therefore, researchers first proposed the optical flow estimation algorithm based on CNN called EchoPWC-Net [22,35]. By calculating the displacement vector changes of pixels in the region of interest in two consecutive frames, EchoPWC-Net quickly and accurately estimated the myocardial motion and fully automatically assessed GLS. Based on 200 cases of 2D echocardiography, the study compared EchoPWC-Net and the commercial semi-automatic software (EchoPAC). The results showed that GLS assessed by the two methods had a highly significant correlation (r = 0.93). First, compared with the sparse speckles of speckle tracking echocardiography, the optical flow estimation algorithm was able to calculate the dense optical flow field of myocardial motion and capture more effective pixel information. Second, the optical flow estimation reduced the post-processing time (single view < 5 s; the average of three views was 13 s), making it possible to measure GLS in real time in the future. In addition, the optical flow algorithm for the fully automated measurement of GLS eliminated intra- and inter-observer variability and had good repeatability. However, the robustness of the model for different image qualities needs to be further evaluated.

Evain E. et al. [36] developed a new optical flow estimation algorithm based on PWC-Net. The results showed that the algorithm accurately assessed GLS in the echocardiography sequences. There was a powerful correlation between automated and manual GLS (r = 0.77). In addition, the study proposed a method to generate simulation datasets (including artifacts or not) to simulate the diversity of datasets in the real world and improve the robustness of the model.

It can be seen that AI algorithms help to improve the accuracy and real-time capability of GLS measurement, thus assisting clinicians in evaluating left ventricular systolic function comprehensively and accurately.

### 4.2. Disease Diagnosis

The strain parameters generated by the post-processing of echocardiography can faithfully reflect myocardial systolic and diastolic deformation, which is helpful for the early diagnosis and prognosis of cardiac dysfunction. However, these high-dimensional data usually contain redundant information, which poses a challenge for data mining and interpretation. Their clinical application is hampered by the lack of feature extraction capability of traditional analysis methods, and hence cannot provide sufficient information for clinicians to make clinical decisions quickly and accurately. This is where AI rises to the occasion. AI has strong feature extraction capability and performs well in analyzing high-dimensional and complex data. It is widely used in the differential diagnosis of diseases.

Narula S. et al. [25] developed an integrated model (SVM, RF, and ANN) with echocardiographic images of 77 patients with physiological myocardial hypertrophy and 62 patients with hypertrophic cardiomyopathy. Combined with strain parameters, the model successfully distinguished the physiological and pathological patterns of myocardial hypertrophy and identified hypertrophic cardiomyopathy with a sensitivity of 0.96 and specificity of 0.77.

Sengupta P.P. et al. [26] conducted a study including 50 patients with constrictive pericarditis, 44 patients with restrictive cardiomyopathy, and 47 controls. Based on the strain parameters, they developed an associative memory classification algorithm and took pathological results as the gold standard. The results showed that the algorithm effectively distinguished constrictive pericarditis and restrictive cardiomyopathy (AUC 0.96).

Zhang J. et al. [27] conducted a study including 217 patients with coronary heart disease and 207 controls. Based on two-dimensional speckle tracking echocardiographic and clinical parameters, they integrated various classification methods by stacking learning strategies to build a prediction model for coronary heart disease. The results showed that the integrated model combined the advantages of multiple classification models. The classification accuracy of coronary heart disease in the test set was 87.7%, the sensitivity was 0.903, the specificity was 0.843, and the AUC was 0.904, which was significantly higher than that of a single model.

Apart from directly using GLS data, researchers also explored disease phenotypes based on strain curves. Loncaric F. et al. [37] conducted a study including 189 patients with hypertension and 97 controls. Based on the strain curve and pulse Doppler velocity curve of mitral and aortic valves, an unsupervised ML algorithm was developed to automatically identify the patterns in the strain and velocity curves throughout cardiac cycles. The algorithm successfully divided hypertension into four different functional phenotypes (P1, healthy; P2, transitional; P3, functional remodeling in response to pressure overload; and P4, related to a higher burden of comorbidities and differences in clinical management in female patients). In addition, Yahav A. et al. [38] developed a fully automated ML algorithm based on strain curves. The strain curve was successfully divided into physiological, non-physiological, or uncertain categories with a classification accuracy of 86.4%.

AI performs well in mining nonlinear characteristic relationships hidden in data, but the lack of interpretability limits its clinical application. Improving the interpretability of AI algorithms and promoting the adaptation of AI-aided diagnosis systems to clinical practice is essential for future research.

**Table 2:** Studies of AI’s Application in Left Ventricular Systolic Function—GLS.

Authors	Year	Task	Models	Dataset	Results
Kawakami H. et al. [34]	2021	GLS assessment	AutoStrain	561 subjects	Automated vs. manual GLS: r = 0.685, bias = 0.99%. Semi-automated vs. manual GLS: r = 0.848, bias = −0.90%. Automated vs. semi-automated GLS: r = 0.775, bias = 1.89%.
Salte I.M. et al. [22]	2021	GLS assessment	EchoPWC-Net	200 studies	EchoPWC-Net vs. EchoPAC: r = 0.93, MD 0.3 ± 0.3%.
Evain E. et al. [36]	2022	GLS assessment	PWC-Net	>60,000 images	Automated vs. Manual GLS: r = 0.77, MAE 2.5 ± 2.1%.
Narula S. et al. [25]	2016	Disease detection (ATH vs. HCM)	Ensemble model (SVM, RF, ANN)	77 ATH, 62 HCM patients	Sensitivity 0.96; specificity 0.77.
Sengupta P.P. et al. [26]	2016	Disease detection (CP vs. RCM)	AMC	50 CP patients, 44 RCM patients, and 47 controls	AUC 0.96.
Zhang J. et al. [27]	2021	Disease detection(CHD)	Two-step stacking	217 CHD patients, 207 controls	Sensitivity 0.903; specificity 0.843; AUC 0.904.
Loncaric F. et al. [37]	2021	Disease detection (HT)	ML	189 HT patients, 97 controls	HT is divided into 4 phenotypes.
Yahav A. et al. [38]	2020	Disease detection (strain curve classification)	ML	424 subjects	Strain curve is divided into physiological, non-physiological, and uncertain categories (accuracy 86.4%).
Pournazari P. et al. [39]	2021	Prognosis analysis (COVID-19)	ML	724 subjects	BC (AUC 0.79). BC + Laboratory data + Vital signs (AUC 0.86). BC + Laboratory data + Vital signs + Echos (AUC 0.92).
Przewlocka-Kosmala M. et al. [40]	2019	Prognosis analysis (HFpEF)	Clustering	177 HFpEF patients, 51 asymptomatic controls	HFpEF is divided into 3 prognostic phenotypes.

GLS, global longitudinal strain; MD, mean difference; MAE, mean absolute error; ATH, athletes; HCM, hypertrophic cardiomyopathy; SVM, support vector machine; RF, random forest; ANN, artificial neural networks; CP, constrictive pericarditis; RCM, restrictive cardiomyopathy; AMC, associative memory classifier; AUC, area under the receiver operating characteristic curve; CHD, coronary heart disease; HT, hypertension; ML, machine learning; HFpEF, heart failure with preserved ejection fraction; BC, baseline characteristics; Echos, echocardiographic measurements.

## 5. Challenges and Future Directions

In recent years, AI-enhanced echocardiography has attracted extensive attention. However, it is in the initial stage of development and still faces many challenges. Firstly, the lack of a large sample and pluralistic and standardized datasets hamper the integration of AI into echocardiographic practice. Secondly, AI-related clinical research is scientifically oriented, requiring every sonographer of the research team to identify and refine vital scientific issues in daily clinical practice, which could be time consuming and infeasible. In addition, traditional ML and DL algorithms have their respective merits and defects. The traditional ML algorithms can be applied to small-sample datasets with certain interpretability, but this does not guarantee exhaustive feature extraction. DL has unique advantages in the data analysis of large samples, but the results lack interpretability, and the sample shortage may lead to model overfitting and limited generalization ability. Therefore, combining traditional ML and DL algorithms might improve the interpretability and sensitivity of intelligent medical prediction models, which is essential for solving critical clinical problems. Additionally, the exploration and application of AI in multimodal imaging are bringing new insights into modern medicine. We are expecting that, in the future, multimodality information such as ultrasound imaging, magnetic resonance imaging, and clinical data could be integrated into a one-stop AI diagnostic model, simplifying clinical diagnoses and the treatment process and improving the detection rate of diseases.

## Data Availability

Not applicable.
